# Towards cholera elimination in Zanzibar: analysis of evidences on what have worked

**DOI:** 10.11604/pamj.supp.2023.45.1.39828

**Published:** 2023-06-14

**Authors:** Ghirmay Andemichael Redae, Tigest Ketsela Mengestu, Fadhil Mohammed Abdalla, Salim Noor Slim, Vendelin Tarmo Simon, Ali Omar Ali, Grace Elizabeth Saguti, Michael Mesfin Habtu, Sisay Gashu Tegegne, Marko John Msambazi, Yoti Zabulon

**Affiliations:** 1 World Health Organization Country Office, Dar es Salaam, Tanzania,; 2 Ministry of Health, Mtoro Rd, Zanzibar, Tanzania,; 3 UNICEF Field Office Zanzibar, Nyerere Rd, Zanzibar, Tanzania

**Keywords:** Cholera elimination, enabling environment, prevention, response, water and sanitation, Oral Cholera Vaccine (OCV), multi-sectoral coordination

## Abstract

Cholera, an enteric disease caused by Vibrio cholera claims thousands of lives yearly. The disease is a disease of inequality that affect populations which have poor access to safe water and sanitation facilities. Zanzibar, an archipelago in the Indian ocean which is part of the United Republic of Tanzania has been affected by recurrent cholera outbreak for the past decades. A multi-sectoral and multi-year three pillar approach namely Enabling Environment, Prevention and Response, for the elimination of cholera were initiated by the stewardship of the government, engagement of the community and technical and financial support of partners. The approach has enabled Zanzibar to interrupt the recurrent cholera outbreak for the past five years. The analysis of evidences have proven that creating an enabling environment through multi-sectoral involvement, mobilizing communities, intensifying surveillance complemented by the traditional disease prevention and control interventions has resulted to interruption of cholera transmission in the country.

## Introduction

Cholera is an acute enteric infection caused by Vibrio cholera through ingestion of the bacterium via contaminated water and food. It is estimated that cholera causes up to four million cases globally and 143,000 deaths yearly; 54% of these are reported from Africa [1,2]. More than 90% of cases and deaths are in less developed countries with poor access to safe water and sanitation [3]. In the 19th century, cholera spread worldwide from its original reservoir in the Ganges delta in India. Six subsequent pandemics killed millions across all continents [1]. The seventh pandemic of cholera that affected all continents reached Africa and Europe in the 1970s. Vibrio cholera inhabit coastal, estuarine and brackish water environments [4]. Four million people in Tanzania have no safe water supply, and 29 million have no sanitation facilities [5]. People living under such circumstances, especially women and children, suffer the most [5].

Zanzibar has had recurrent outbreaks of cholera since 1978. Seventeen cholera outbreaks were recorded, with 14,364 cases and 210 deaths. The most recent major cholera outbreak lasted 10 months between September 2015 to July 2016, recording 4,330 cases and 68 deaths, with a case fatality rate of 1.6% [6]. In cognizance of the health and economic impacts of repeated cholera outbreaks in the islands, the Ministry of Health and several sectors under the leadership of the Second Vice President Office developed a 10-year multisectoral plan to eliminate cholera, Zanzibar Comprehensive Cholera Elimination Plan (ZACCEP) 2018-2027 [6]. The elimination plan has three pillars: enabling environment, prevention, and response. Enabling environment: for cholera to be eliminated, there is a need for multisectoral coordination, the building of capacity of the sectors, implementation of public health rules and regulations on food hygiene, safe water supply and sanitation, enhancing surveillance capacity, continuous risk assessment and monitoring and evaluation of the elimination agenda. All these are addressed under enabling environment. Prevention: the second pillar for the elimination of cholera is prevention, under which ensuring safe water supply, sanitation infrastructures, social and behavioral change communication and provision of oral cholera vaccine are implemented. Response: the third pillar focuses on case management and ensuring that logistics and supplies are continuously available if a cholera outbreak occurs.

Essential medicines and supplies were pre-positioned in every district, and cholera beds were stored at the disaster management committee to be deployed whenever needed. In 2017, the World Health Organization (WHO) launched a roadmap for ending cholera by 2030, targeting to reduce cholera deaths by 90% and eliminate the disease from at least 20 endemic countries. The road map uses three strategies: early detection and quick response, targeted prevention, support resources, and partnership [7]. The African Development Bank (AfDB) invested 40.9 million USD in improving Zanzibar’s water and sanitation infrastructures [8]. According to the Government of Zanzibar report, more than 123 million US Dollars was invested in improving water and sanitation infrastructure in Zanzibar from 2018-2020. This paper aims to share best practices of controlling cholera in Zanzibar which was due to multisectoral involvement and partnership, improved access to safe water supply and provision of oral cholera vaccine as a supplemental public health intervention.

## Methods

We conducted a retrospective analysis of the implementation of proven interventions as stipulated in the strategic document for 2018-2027 based on the three pillars identified for eliminating cholera in Zanzibar by 2027. Zanzibar, an archipelago of 46 islands, two of them being the major islands in terms of size and population, was the target for cholera elimination. The entire population of Zanzibar was targeted for cholera elimination with the implementation of specific interventions, such as the cholera vaccine for 322,483 people in cholera hotspot areas in both Unguja and Pemba.

**Data collection tool and analysis:** policy and strategic documents, Integrated Disease Surveillance and Response (IDSR) data and Oral Cholera Vaccine (OCV) registries were monitored and analyzed. Post campaign coverage survey was conducted, and data was analyzed on Water, Sanitation and Hygiene (WASH) and vaccination coverage.

**Processes for program development for cholera elimination in Zanzibar:** the highest office in Zanzibar, the president’s office, instructed all relevant sectors to work together and control the cholera outbreak of 2015-2016 and develop a multisectoral and multi-year cholera elimination plan. The instruction was communicated to key sectors that will play a major role in cholera control and elimination. The Ministry of Health was selected to be the technical arm of the initiative and requested WHO and other development partners for technical and financial support. A task force was formed composed of technical officers from MOH, Ministry of Water and Natural Resources, represented by Zanzibar Water Authority (ZAWA), Local Government/Municipality, UN agencies, and other development partners and NGOs. The task force had clear terms of reference and had regular meetings until the cholera elimination plan was drafted, a consensus-building meeting was done, and the Zanzibar Comprehensive Cholera Elimination Plan (ZACCEP) 2018-2027 was launched. The process was guided by the Second Vice President’s Office and MOH as a technical arm from the government side, and WHO as a coordinating agency for development partners.

## Results

**Figure 1:**
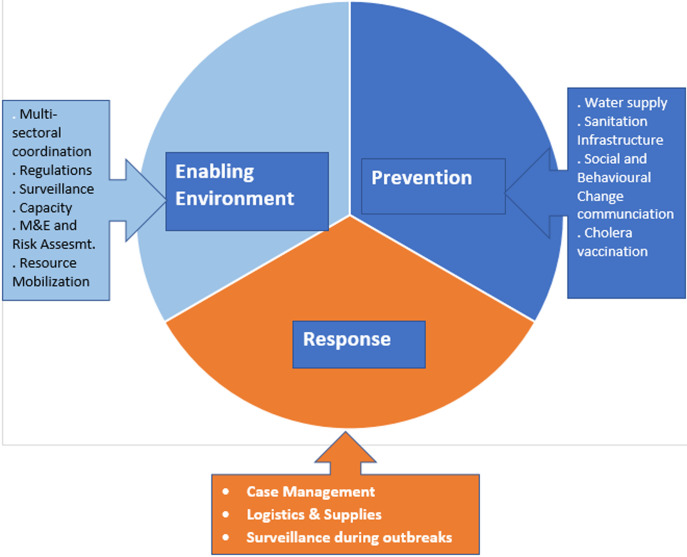
three pillars of cholera elimination for Zanzibar: enabling environment, prevention and response

As indicated in the ZACCEP document, the implementation of activities and interventions was closely monitored through the coordination structures set out during the inception of the three pillars for eliminating cholera in Zanzibar (Figure 1).

**Figure 2:**
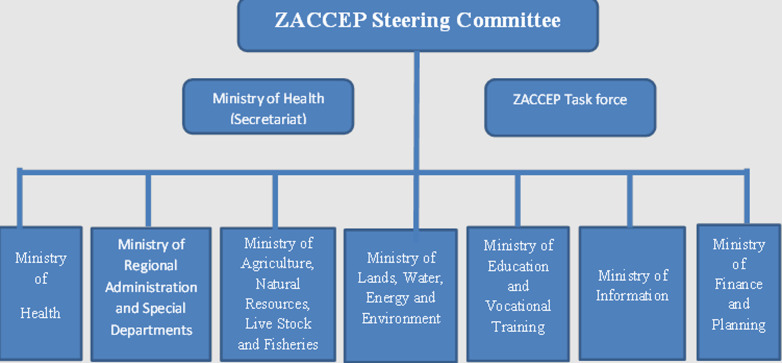
cholera coordination mechanisms/structure in Zanzibar recommended in 2018

**Enabling environment**: the Revolutionary Government of Zanzibar has established a coordination mechanism for the cholera elimination agenda in Zanzibar. The coordination is led by the Second Vice President Office (SVPO). A high-level steering committee was established, composed of Principal Secretaries of all sectors and a task force that monitors the implementation of ZACCEP and reports to the steering committee for policy and strategic guidance. The steering committee was integrated into the existing Principal Secretaries coordination body that meets every week; however, it meets biannually for cholera and other emergency-related issues. The task force meets quarterly and on Ad-hock bases when required. This has been a very instrumental mechanism for multiple sectors and partners to work together to implement ZACCEP effectively (Figure 2). The coordination mechanism was also cascaded to regional and district levels, mainly guided by Regional and District Medical Offices under the strong guidance and support of Regional and District administrative bodies. The strong coordination mechanism has contributed to eliminating cholera in Zanzibar.

Public health regulations on food hygiene and sanitation were enforced through advocacy, orientation and training to 4,000 food handlers, providing them with gowns, hair covers and other amenities. Food and drink establishments that do not adhere to public health safety were given written warnings and measures up to closure if they did not comply. In addition, more than 1,200 health workers, point of entry and staff from various sectors and 4,000 food handlers were trained on cholera from 2018-2022. Additional 400 vaccinators, district medical officers, village authorities and more than 1,700 community volunteers were also trained and engaged in oral cholera vaccination

**Prevention:** through loans and grants, the Revolutionary Government of Zanzibar mobilized more than 146 million US$ for water and sanitation infrastructure improvement. The resources were used to construct three water tanks and improve sanitation in cholera hotspot slums. The African Development Bank (AfDB), through the Urban Water and Sanitation Project, provided 40,944,216 US$ for water and sanitation infrastructure improvement in Zanzibar [8]. Under the coordination of the Health Promotion unit of the Ministry of Health, WHO, UNICEF, Red Cross Society of Tanzania, Pemba Island Relief Organization (PIRO), and many other partners have supported community sensitization, education and awareness creation. These were done using a house-to-house strategy, using mobile vans and various media, including television and radio, which were consistently aired during outbreaks of cholera and beyond.

**Figure 3:**
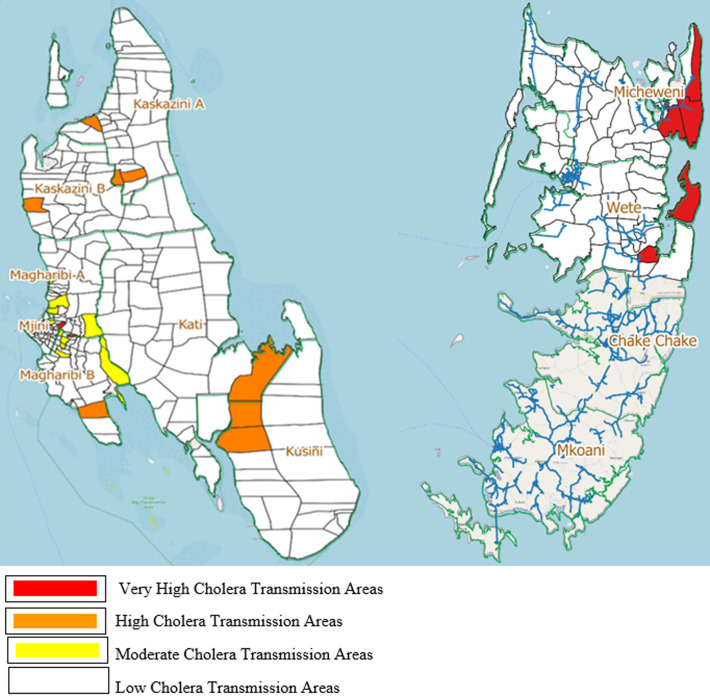
intensity of cholera hotspots in Zanzibar targeted for oral cholera vaccine August 2021

Chlorine tablets for household water treatment were distributed during community education and sensitization sessions. Three water reservoirs with more than 3 million liters capacity were built, and chlorination was done in these reservoirs before distribution for consumption. The water pipe system, which was old, and leaking, was changed in urban areas where recurrent cholera outbreaks were common. Two rounds of oral cholera vaccinations were conducted in July and August 2021 amid the COVID-19 pandemic targeting 322,483 people. A total of 295,849 (91.7%) individuals above 1 year of age were vaccinated in the first round and 188,354 (63.7%) in the second round. A total of 33 villages from nine districts were selected as cholera hotspots targeted for oral cholera vaccine and other public health measures, including water and sanitation improvement and social mobilization interventions. The target population was selected based on the intensity and recurrence of cholera outbreaks in the past (Figure 3, Table 1).

**Table 1:** oral cholera vaccine (OCV) coverage by districts, Zanzibar 2021

		First round OCV		Second round OCV	
District	Target	Vaccinated	% round-1	Vaccinated	% round-2
**Urban**	64,130	60,714	94.7	46,340	76.3
**West A**	98,139	97,066	98.9	60,114	61.9
**West B**	114,181	95,747	83.9	55,573	58.0
**North A**	8,397	6,652	79.2	4,238	63.7
**North B**	2,703	2,435	90.1	1,605	65.9
**Central**	1,026	924	90.1	808	87.4
**South**	3,126	2,808	89.8	2,229	79.4
**Wete**	7,002	9,547	136.3	5,463	57.2
**Micheweni**	23,779	19,956	83.9	11,984	60.1
**TOTAL**	**322,483**	**295,849**	**91.7**	**188,354**	**63.7**

**Figure 4:**
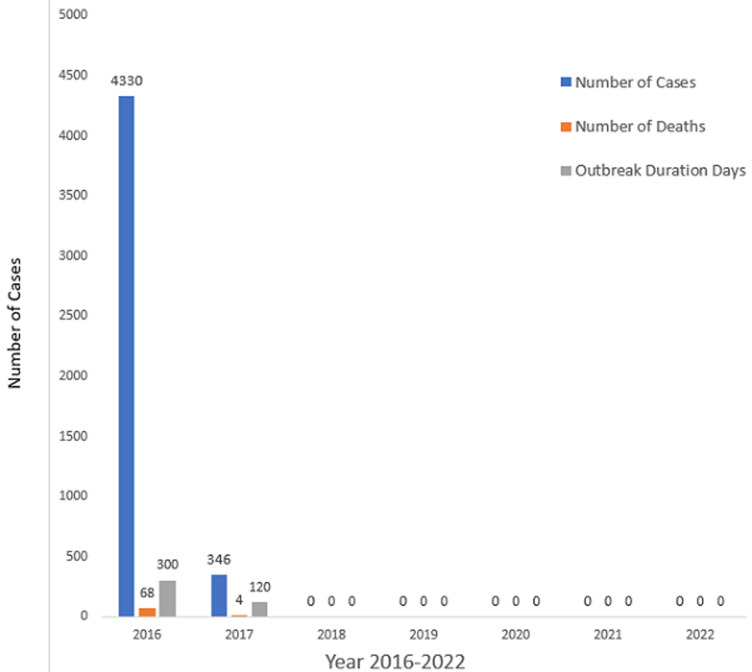
number of cholera cases by year, number of cases, deaths and duration of cholera outbreak in days, Zanzibar 2016 to 2022

**Response:** to prepare for outbreaks, the Ministry of Health pre-positioned a set of intravenous fluids, oral rehydration solutions, chlorine tablets and antibiotics in every district for immediate response in case a cholera outbreak happens. The disaster risk management directorate at Vice President’s Office stocked tents, cholera beds, buckets for chlorine preparation and other amenities ready to be deployed in case of cholera outbreaks and other health emergencies. Because of the successful implementation of interventions across the three pillars of cholera elimination, namely Enabling Environment, Prevention, and Response, Zanzibar has not been reported since 2017. Significant progress has been made in Zanzibar since the cholera outbreak five years ago. The improvement of water and sanitation infrastructures, coupled with a comprehensive plan to eliminate cholera, has set Zanzibar on the path towards a future free from this disease and contributing to elimination of cholera in the United Republic of Tanzania (Figure 4). Application for certification of eliminating cholera will be done by the United Republic of Tanzania and processes recommended by WHO will be followed for certification.

## Discussion

The three pillars approach for eliminating cholera is a prototype that Zanzibar adopted, including enabling environment, prevention and response. It was found effective in interrupting the recurrent cholera outbreak in Zanzibar. Other countries traditionally implement water, sanitation, and hygiene improvement, treatment of cases and oral cholera vaccines for interrupting cholera transmission in their respective countries. However, Zanzibar has added an enabling environment that invested in multisectoral coordination, implementation of rules and regulations related to food hygiene and sanitation and capacity building of sectors in responding to cholera outbreaks.

Similarly, Zambia initiated a multisectoral action to control Lusaka’s cholera outbreak, coordinated by Zambia National Public Health Institute in 2018 [9]. Haiti also invested in improving water and sanitation infrastructures after the cholera outbreak that followed a massive earthquake that claimed nearly 10,000 lives and caused more than 820,000 cases [10]. Reactive cholera vaccination was conducted through coordination with the Ministry of Public Health and Population of Haiti [11]. Similarly, Zimbabwe engaged in prevention and response activities after it experienced an unprecedented cholera outbreak in 2008 and 2009, with a total reported cases of 98,592 and 4,288 deaths [12]. Amidst economic and systems challenges, Zimbabwe developed a multisectoral cholera elimination plan under the leadership of the Vice President and Minister of Health, a hybrid of the Zanzibar approach [13]. In Zanzibar, the Second Vice President’s Office has assumed the coordinated role as per its mandate for disaster risk and public health emergencies [14]. The cholera vaccination coverage was very high for the first round, while the second round plunged by 28% low. According to the anecdotal interview with parents and caregivers, the reasons for low coverage during the second round were attributed to the launching of the COVID-19 vaccination. Communities were suspicious that the vaccine could be COVID-19 and not cholera.

We also note that the OCV coverage in Cameroon, a reactive vaccination during COVID-19, had a similar coverage of 64.4% [15]. A door-to-door approach in the Mogede District of Cameroon, targeting 126,619 population above 1 year of age, has attained 81.0% and 80.1% OCV coverage in the first and second rounds, respectively [16]. The study in Zimbabwe has revealed that using a highly effective vaccine with reasonable coverage of at least 58% and efficacy of 75% can prevent cholera outbreaks through reproductive factors of 1.82 [17]. Hence the high OCV coverage in Zanzibar has provided an additional public health shield to protect the people from cholera.

Using community volunteers and hygiene promoters to give lifesaving information on water and sanitation effectively reduced the spread of cholera in Yemen [18]. Despite the massive engagement of communities and community volunteers in Zanzibar, their role is not well documented. The sword and shield approach in West Africa, a sword for responding to outbreaks and a shield for investing in integrated safe water supply and sanitation infrastructure during calm periods, which are elements of the three pillar approach in Zanzibar, contribute to the reduction of cholera outbreaks in west African countries [19]. Continuous community sensitization, engagement and participation before and during the implementation of interventions such as the provision of cholera vaccination and innovative approaches such as door-to-door approach could improve vaccination coverage and is highly recommended.

Implementation of investment in water and sanitation is the primary and sustainable way of preventing cholera and other diarrheal diseases transmitted through contaminated food and water [20]. The Government of Zanzibar has invested in water and sanitation infrastructures in the underserved populations previously affected by recurrent cholera outbreaks. Proper mapping, identifying hotspots, and implementing public health measures has proven effective in controlling and eliminating cholera in Zanzibar. It was noted that when new vaccines, like COVID-19, are concomitantly availed to the community, they can compromise the coverage, as was observed in Zanzibar. We recommend that high-level government commitment and leadership demonstrated with allocating resources for eliminating cholera in Zanzibar is exemplary that other countries could adopt. Implementing the Paris Declaration on aid effectiveness, with particular emphasis on harmonizing partners´ support to the government’s strategic vision, has proven to facilitate the implementation of interventions for cholera elimination.

The lessons learned from cholera can be replicated in controlling and eliminating other diseases of public health concern in the context of the One-Health approach. Continuous community sensitization, education and engagement and documenting their contributions towards cholera elimination are recommended to sustain gains and consolidate the elimination of the disease.

## Conclusion

We conclude that implementing the three pillars, namely enabling environment, prevention, and response, under the coordination of the Second Vice President’s Office has proven to be very effective in controlling and eliminating cholera in Zanzibar. Implementation of investment in water and sanitation is the primary and sustainable way of preventing cholera and other diarrheal diseases transmitted through contaminated food and water [20]. The Government of Zanzibar has invested in water and sanitation infrastructures in the underserved populations previously affected by recurrent cholera outbreaks. Proper mapping, identifying hotspots, and implementing public health measures has proven effective in controlling and eliminating cholera in Zanzibar. It was noted that when new vaccines, like COVID-19, are concomitantly availed to the community, they can compromise the coverage, as was observed in Zanzibar. We recommend that high-level government commitment and leadership demonstrated with allocating resources for eliminating cholera in Zanzibar is exemplary that other countries could adopt. Implementing the Paris declaration on aid effectiveness, with particular emphasis on harmonising partners´ support to the government’s strategic vision, has proven to facilitate the implementation of interventions for cholera elimination. The lesson learnt from cholera can be replicated in controlling and eliminating other diseases of public health concern in the context of the One-Health approach. Continuous community sensitisation, education and engagement and documenting their contributions towards cholera elimination are recommended to sustain gains and consolidate the elimination of the disease.

### What is known about this topic

- Alignment of the various strategic visions, missions and priorities is crucial for the success of strategic plans;
- Stakeholder engagement and participatory approach right from the inception of the concept in strategic plan to the finalization of the plan is critical in realization of goals and objectives as well as underlining accountability of each actor in the plan;
- The CCS has been a midterm strategic vision for WHO to support member states.

### What this study adds

- The methods that we used in meticulous review of documents in the prioritization of interventions and focus areas, and the mapping of various strategic priority areas for alignment would help countries that will undergo similar process to follow. In addition, this helped in aligning the monitoring and evaluation framework of the CCS with the UNSDCF and the national health sector strategic plan.
